# Understanding Daily, Emotional, and Physical Burdens and Needs of Parents Caring for Children with Type 1 Diabetes

**DOI:** 10.1155/2022/9604115

**Published:** 2022-12-13

**Authors:** Heike Saßmann, Su-Jong Kim-Dorner, Verena Berndt, Torben Biester, Andrea Dehn-Hindenberg, Bettina Heidtmann, Norbert Jorch, Eggert Lilienthal, Nicole Nellen-Hellmuth, Andreas Neu, Katja Schaaf, Ralph Ziegler, Karin Lange

**Affiliations:** ^1^Hannover Medical School, Medical Psychology, Hannover, Germany; ^2^Sana Hospital Group Berlin-Brandenburg, Social-Pediatric Centre Lichtenberg, Berlin, Germany; ^3^Children's Hospital AUF DER BULT, Diabetes-Centre for Children and Adolescents, Hannover, Germany; ^4^Catholic Children's Hospital Wilhelmstift, Hamburg, Germany; ^5^Bielefeld University, University Clinic for Pediatrics, Evang. Klinikum Bethel, Bielefeld, Germany; ^6^University Clinic Ruhr-University Bochum, University Children's Hospital, Bochum, Germany; ^7^Leopoldina-Hospital Schweinfurt, Clinic for Pediatrics, Schweinfurt, Germany; ^8^Eberhard Karls University Tübingen, Pediatric Endocrinology and Diabetes, Tübingen, Germany; ^9^Elisabeth-Hospital-Essen, Pediatrics, Essen, Germany; ^10^Diabetes Clinic for Children and Adolescents Münster, Münster, Germany

## Abstract

**Aims:**

To investigate (1) daily, emotional, and physical caregiving burdens in parents of children with type 1 diabetes, (2) the sociodemographic and clinical predictors of three burdens, and (3) support measures that parents wish to receive.

**Methods:**

The study was a multicenter cross-sectional survey conducted in nine German pediatric diabetes centers. A questionnaire assessing three types of burdens and wishes for support was distributed to parents with a child with type 1 diabetes visiting one of the pediatric centers for a routine check-up.

**Results:**

Data from 1,107 parents (83% mothers) were analyzed. Parents reported significantly higher emotional burdens compared to daily and physical burdens (*p* < 0.0001). Mothers felt more burdened than fathers did. Parents of younger children reported higher daily and physical burdens compared to the parents of older children, and similarly, parents of technology users reported higher daily and physical burdens compared to the parents of nontechnology users. However, emotional burdens did not differ in both comparisons. Other demographic factors (i.e., parent's age, migration status, and single-parent family status) predicted high levels of daily or physical burdens, but only HbA1c level and the parent's gender (mother) predicted a high emotional burden. Independent of the level of burden, 78% of parents wanted additional diabetes training.

**Conclusion:**

Despite parents reporting high emotional burdens in connection with diabetes care, HbA1c and the gender of the reporting parent were the only risk factors. As the child gets older, parents' daily and physical distress decrease but not the emotional burden. Diabetes training including regularly offered booster sessions as well as low-threshold interventions for mental health issues and practical self-care skills is recommended to provide continuous support for parents.

## 1. Introduction

Successful self-management of type 1 diabetes (T1D) requires multiple tasks and responsibilities. For very young patients, sole responsibilities for daily diabetes management fall upon parents while they simultaneously perform the general tasks of parenting [1–4]. These additional tasks of managing T1D in a child place a substantial load on parents, which is often linked to diabetes distress and mental health problems among parents [4–6]. Approximately 20-30% of parents report diabetes distress with depression and stress symptoms related to managing a child with T1D [4, 7–9]. In a semistructured interview study with parents of 79 children with T1D, the parents mentioned that the emotional burden was the most pressing theme of caring for their children with diabetes [5].

These diabetes burdens in parents are detrimental to parental health and often directly and indirectly linked to their children's diabetes management, metabolic control, distress, and quality of life [7, 9–12]. Family functioning and treatment adherence are directly associated with metabolic control in the child with diabetes [7, 10–16]. In a sample of U.S. mothers of teens with T1D, diabetes distress was strongly related to maternal depression, relationship distress, and glycemic control [9]. A web-based survey in Denmark also confirmed that family functioning such as problem-solving and affective responsiveness was associated with HbA1c levels in adolescents with diabetes [16]. Furthermore, reduced parental well-being is associated with increased problem behaviors in children, which is in turn negatively related to glycemic control [17]. That is, unsupportive parenting behaviors and parental diabetes distress were related to inferior glycemic control in a sample of Dutch parents and their youth [17].

Several other demographic risk factors were also identified for increased stress and strains in parents of children with T1D. A high level of burden was reported among parent-caregivers who had young children and low education and were unemployed in a Polish study [18]. Parents' gender was also linked to diabetes distress. Despite both parents reporting distress associated with managing T1D in their child, higher rates of distress were reported by mothers [19, 20]. Considering mothers are often the primary caregivers for the child with T1D [21], providing necessary and appropriate support is contingent upon understanding the types and extent of burdens that mothers report.

Moreover, recent studies described that the use of diabetes technologies such as pump therapy (continuous subcutaneous insulin infusion (CSII)) and continuous blood glucose monitoring (CGM) systems is associated with lower parental burdens. That is, the use of new technologies is associated with reduced fear of hypoglycemia, better nocturnal control, greater comfort with other caregivers due to the use of remote control, and better glucose control [6, 22, 23]. However, it is also associated with new challenges and tasks in everyday life such as alerts, CGM data interpretation, insertions of the sensor, and multiple device placements on a child's small body [22]. Clearly, the connections between the parental burden and sociodemographic characteristics in conjunction with diabetes technology use need further investigation.

In the current study, we aimed to examine the different types of burdens, namely, daily, emotional, and physical, that parents' experience and the influence of sociodemographic and clinical characteristics on these burdens. Moreover, we examined the ways in which parental burdens can be reduced by assessing diabetes support measures that parents wish to receive. Understanding parental diabetes burdens coupled with associated parameters and desired assistance will help to provide adequate support to alleviate distress in parents and increase the well-being of the entire family living with a child with diabetes.

## 2. Materials and Methods

This study was part of a larger cross-sectional research project, the AMBA study (Alltagsbelastungen der Mütter und Väter von Kindern mit Typ 1 Diabetes: Auswirkungen auf Berufstätigkeit und Bedarf an Unterstützung im Alltag—translation: Everyday stress of mothers and fathers of children with type 1 diabetes: effects on employment and need for support in everyday life), where the impact of having a child with diabetes in the family was examined [21]. In our sampling method, study sites were selected based on the center's qualification, geographical location, and the size of the cities. Nine pediatric diabetes centers certified by the German Diabetes Association were invited to participate in the study: Sana Hospital Group Berlin-Brandenburg, Social-Pediatric Centre Lichtenberg, Berlin (*n* = 116); Bielefeld University, University Clinic for Pediatrics, Bielefeld (*n* = 88); University Clinic Ruhr-University Bochum, University Children's Hospital, Bochum (*n* = 107); Elisabeth-Hospital-Essen, Pediatrics, Essen (*n* = 49); Children's Hospital Auf der Bult, Hannover (*n* = 299); Catholic Children's Hospital Wilhelmstift, Hamburg (*n* = 194); Diabetes Clinic for Children and Adolescents Münster, Münster (*n* = 141), Leopoldina-Hospital Schweinfurt, Clinic for Pediatrics, Schweinfurt (*n* = 36), and Eberhard Karls University Tübingen, Pediatric Endocrinology and Diabetes, Tübingen (*n* = 77). Each center has a meaningful catchment area including families from urban and rural districts. Two sites are in large cities with >1 million residents; two sites are in small cities with <100.000 residents. Centers from eastern, northern, western, and southern parts of Germany were included. Centers were treated as a unit, and each eligible family cared for in each center was invited to participate. We wanted to gain as much information as possible, especially on small subgroups, e. g., single parents. Therefore, we chose to invite the entire eligible population of each center. The data were collected during a three-month period between January and June 2018. As quarterly in-person appointments are the standard of pediatric long-term care in the German health care system, the whole center population with diabetes was seen within the recruiting period.

### 2.1. Participants

Mothers and fathers of children diagnosed with T1D were recruited during a routine quarterly check-up visit to one of the nine pediatric diabetes centers. The inclusion criteria of the parents were as follows: child's age at diagnosis ≤ 14 years because the responsibility of diabetes treatment is placed mainly on the teen starting at age 15 [24], diabetes duration of at least 12 months to reduce the potential impact of additional stress and partial remission due to new diagnosis [24], and the ability to comprehend verbal and written German to provide verbal consent and complete the questionnaire. Data were excluded if the questionnaire was completed by someone other than the mother or father, none of the three burden measures were completed, or the participating parent was not currently living with the child with diabetes at the time of the study to assess current caregiving burdens. Following the study aim, an a priori sample calculation was not performed but instead, optimizing the available resources during the allotted three-month data collection period; each and every family visiting the diabetes center was invited to participate in the study.

### 2.2. Procedures

Eligible families were approached by in-clinic solicitation while in the waiting area. All participants provided verbal consent upon receiving information on the purposes, procedures, and anonymous data collection process. Only one parent per family self-administered the questionnaire. In case both parents were present, they were asked to choose one parent to complete the questionnaire alone. The participants returned the completed questionnaire in a mailbox in the waiting area upon departure. A prepaid envelope addressed to the study center was also provided if the participant wanted to take the questionnaire home. In such cases, participants were told to post the envelope within the next two days. Verbal instructions were given to parents to answer all questions without skipping an item. All complete and incomplete questionnaires collected at the diabetes centers were sent to Hannover Medical School (MHH) where data entry and evaluation were performed. Paper questionnaires are stored in a secure area and kept for the following 5 years. The study procedures were approved by the Ethical Committee on Human Studies at MHH (no. 3689-2017) in accordance with the Declaration of Helsinki.

### 2.3. Measures

The current questionnaire was developed based on a previous survey, conducted in 2003 assessing the living conditions of families with a child T1D [25]. For the current study, demographic and clinical characteristics, assessments on self-reported parental burdens, and diabetes support items were used. The demographic and clinical characteristic portion of the questionnaire has been already published [21]. The demographic variables included participating parents' characteristics (gender, education, age, number of children, living arrangement, and immigrant status) and the child's age, gender, and clinical characteristics (HbA1c, disease duration, and diabetes technology use). For this study, patients using CSII, CGM, or both were defined as “technology users.”

Three types of parental burdens (daily, emotional, and physical) were examined by asking parents to quantify their strains due to the child's diabetes care. The answers were provided by using a 5-point Likert scale of “very low” (1), “low” (2), “moderate” (3), “high” (4), and “very high” (5). They were also asked if they had been diagnosed with any psychological disorders since the onset of diabetes in their child. Parents' wishes and needs for diabetes support measures were examined using a list of 8 support offers (i.e., diabetes training, self-help patient group, diabetes nannies, web-based consultation, websites for parents of children with diabetes, care service, home office/work from home opportunities, and flexible working hours). Parents indicated whether they are likely to use these support measures if offered.

### 2.4. Data Analyses

Data analyses were conducted by using the statistical software package IBM SPSS Version 27.0 (SPSS Inc., Chicago, Illinois); and Excel (Microsoft Office Professional Plus 2016) was used to create figures. In all analyses, missing variables were handled by using the pairwise deletion method for analyses to maximize all data available. Accounting for the large sample size and multiple comparisons, the statistical significance was set at *p* < 0.01 (two-tailed) for all analyses. However, *p* < 0.05 (two-tailed) was considered significant for logistic regression analyses due to its exploratory nature.

The participant characteristics are presented as means (M) and standard deviations (SD) or absolute numbers and percentages and compared with independent *t*-tests for continuous and *χ*^2^ tests for categorical variables. Three burden data are presented by using frequencies, median (Mdn), and M ± SD to provide a complete picture of reported burdens and analyzed by using nonparametric tests because they are measured in ordinal scale: Mann–Whitney test, Kruskal-Wallis, and Friedman's ANOVA.

Three separate exploratory logistic analyses using the backward stepwise method were performed for daily, emotional, and physical burdens to examine the potential variables associated with high burdens among parents. For logistic analyses, the burden data were dichotomized as low- (“very low,” “low,” and “moderate” responses) and high-burden (“high” and “very high”) and used as the outcome variable. The independent variables were participating parents' sociodemographic characteristics (gender, education, age, number of children, living arrangement, and immigrant status), the child's age, and clinical characteristics (HbA1c, disease duration, and diabetes technology use). Finally, parents' responses to diabetes support are presented for all parents and separately by the low- and high-burden category groups as mentioned above. The low- and high-burden groups were compared by performing *χ*^2^ analyses.

## 3. Results

### 3.1. Study Population

Out of 1,470 eligible families in the nine centers, 1,192 (81% response rate) agreed to participate and completed the questionnaire. Of the 1,192, 48 participants' questionnaires could not be used due to a large number of missing data. Further 37 participants' data were excluded following the inclusion and exclusion criteria. The characteristics of the final sample of 1,107 parents are presented in Table 1. The majority of respondents were mothers (83.0%), living with a spouse (81.1%), and were born in Germany (84.5%). The mean age of participating parents was 44.2 (SD: 6.7) years. Their children had a mean age of 12.7 (SD: 3.9) years with a mean disease duration of 5.9 (SD: 3.8) years, and 501 (46.7%) children were girls. Approximately 56% of preadolescent (age < 13 years) and 41.4% of adolescent children (≥13 years) achieved the 2019 treatment target of HbA1c < 7.5% (58 mmol/mol) [26].

Parents' gender or education did not make a difference in children's HbA1c levels, but the children of German-born parents had lower scores of HbA1c compared to the children of foreign-born parents (M (SD) = 7.6(1.1)%/59.7(12.1) mmol/mol and 8.0(1.3)%/63.4(14.2) mmol/mol, respectively, *t*(970) = −3.32, *p* < 0.001). The distribution of therapeutic regimens as CSII and CGM in this sample was comparable to that of the published data from the German Diabetes Prospective Follow-up registry (Diabetes Patienten Verlaufsdokumentation (DPV)) [26–28]; thus, in terms of diabetes technology use, our sample was representative of typical families with a child with diabetes. The children from German-born parents used CSII significantly more frequently than the children from non-German-born parents (67.2% and 55.0%, respectively, *χ*^2^(1) = 9.41, *p* = 0.002), and CGM use was marginally higher (German-born: 31.1% vs. non-German-born: 19.8%, *χ*^2^(1) = 5.04, *p* = 0.025). HbA1c levels did not differ by CSII use but were lower among CGM users compared to non-CGM users (M(SD) = 7.4(0.8)%/57.4(8.4) mmol/mol and 7.7(1.3)%/60.8(14.2) mmol/mol, respectively, *t*(589) = 2.91, *p* = 0.004).

### 3.2. Daily, Emotional, and Physical Burdens in Parents

**Figure 1:**
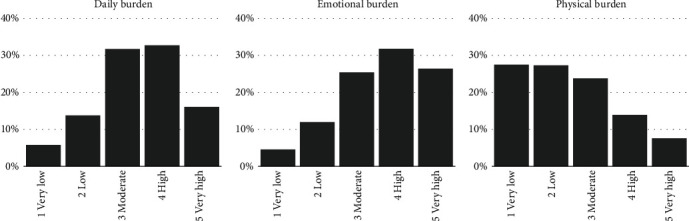
Frequency distribution of daily, emotional, and physical burdens reported by German parents living with a child with type 1 diabetes. Note: daily burden (*n* = 1,093): Mdn = 3 (moderate) and M(SD) = 3.40 (1.09); emotional burden (*n* = 1,096): Mdn = 4 (high) and M(SD) = 3.63 (1.13); physical burden (*n* = 1,086): Mdn = 2 (low) and M(SD) = 2.47 (1.24).

Of the 1,107 parents, 1,067 parents completed all three burden measures, 34 completed two, and six completed one. Parents reported a “high” level of daily burden most frequently (32.7%) followed by a “moderate” level (31.7%) (Figure 1). Concerning the emotional burden, 31.8% reported a “high” level followed by 26.4% having a “very high” level. In contrast, for physical burden, parents reported “very low” and “low” levels with a frequency of 27.5% and 27.3%, respectively. Parents reported the emotional burden to be the highest, followed by daily, and physical burdens due to their child's diabetes (all comparisons significant at *p* < 0.0001). No significant differences in three burdens were found among the nine study sites (daily: Kruskal-Wallis *H* = 10.26 (8), emotional: *H* = 15.44 (8), physical: *H* = 7.79 (8), all *p* = ns).

#### 3.2.1. Association of Three Burdens with Demographic and Clinical Features

Mothers reported significantly higher daily and emotional burdens and marginally higher physical burden compared to fathers (*Z* = −5.76, *p* < 0.001; *Z* = −5.90, *p* < 0.001; and *Z* = −2.26, *p* = 0.024, respectively) (see Table 2). Due to a large sample size difference between mothers and fathers accompanying their children to the clinic, a randomly selected small group of mothers (*n* = 200) was compared to fathers (*n* = 188) as a sensitivity analysis to verify the results of the primary analysis. The results confirmed the findings of primary analysis (daily: *Z* = −5.44, *p* < 0.001; emotional: *Z* = −4.36, *p* < 0.001; and physical: *Z* = −2.37, *p* = 0.018). The living arrangement made a significant difference in physical burdens. That is, the participating parents from two-parent households reported significantly lower physical burdens compared to the other family settings (*Z* = −3.23, *p* = 0.002). A randomly selected small group of parents living with their spouses (*n* = 220) was compared to single parents (*n* = 209) as a sensitivity test. The results also confirmed the findings of primary analysis (daily: *Z* = −1.30, *p* = 0.19; emotional: *Z* = −.97, *p* = 0.33; and physical: *Z* = −2.64, *p* = 0.008). On all three types of burdens, mothers of children in one-parent households reported the highest levels of burdens, and fathers of children living with both parents reported the lowest levels of burdens. Mothers from one-parent households reported a significantly higher level of physical burden (*Z* = −2.99, *p* = 0.003) than mothers living with a spouse.

A total of 132 mothers (14.5%) and 11 (5.9%) fathers reported that they were diagnosed with at least one psychological disorder since their child's diabetes onset. Depression (8.5%) followed by anxiety (3.6%) was most commonly reported among mothers. Parents reporting “high” or “very high” levels of burdens had more frequent diagnoses of at least one psychological disorder compared to parents reporting “very low” to “moderate” levels: 20.1% vs. 6.4% for daily, 19.0% vs. 4.8% for emotional, and 26.2% vs. 9.4% for physical burdens, respectively (all comparisons significant *p* < 0.0001).

Having a young child made a difference in daily and physical parental burdens. Daily and physical burdens were significantly higher for parents of preadolescents (< 13 years) compared with parents of adolescents (daily: *Z* = −6.40, *p* < 0.001 and physical: *Z* = −5.98, *p* < 0.001) (see Table 2). However, the emotional burden did not differ between the parents of the two age groups (*Z* = −1.67, *p* = ns).

Parents of technology users, defined as using either CSII, CGM, or both, reported significantly higher daily and physical burdens compared to parents of nontechnology users (*Z* = −4.88 and *Z* = −4.01, both *p* < 0.0001, respectively) (see Table 2). However, the use of technology did not make a difference in the emotional burden reported by parents (*Z* = −1.58, *p* = ns). A randomly selected small group of parents whose children were diabetes technology users (*n* = 230) was compared to the parents of nontechnology users (*n* = 221). The results confirmed the findings of primary analysis (daily: *Z* = −3.63, *p* < 0.001; emotional: *Z* = −1.76, *p* = 0.078; and physical: *Z* = −3.11, *p* = 0.002).

#### 3.2.2. Predictors of Three Parental Burdens

When dichotomized, 48.8% of parents were categorized as the high daily burden group, 58.0% as the high emotional burden group, and 21.5% as the high physical burden group. The results from three separate logistic regression analyses are presented in Table 3. Mothers were 2.5 to 3 times more likely to be in the high-burdens categories than fathers. An increase in HbA1c and parent's age and the use of technology were predictors of high daily burden, whereas having an immigrant status, increasing parent's age, single-parent status, and the use of diabetes technology were predictors of high physical burden. In contrast, the increasing age of the child reduced the probability of reporting high daily and physical burdens. For high emotional burden, in addition to the parent's gender (mother), the child's increasing values of HbA1c were the only significant predictor.

### 3.3. Parents' Wishes and Needs of Assistance

Of the 1,014 parents who completed the diabetes support portion of the survey, the majority of parents (78%) expressed that they would like to receive additional diabetes training followed by flexible working hours for parents (61%) and websites for parents of children with diabetes (53%) (Table 4). Mothers asked for self-help/patient groups and nanny services more frequently than the fathers did (25.1% vs. 14.4%, respectively, *p* = 0.003 and 39.3% vs. 28.1%, respectively, *p* = 0.006), but both asked for all other suggested assistance measures relatively equal. Parents of preadolescents asked for support more frequently than the parents of adolescents (all significant at *p* ≤ 0.002) except for the self-help/patient group and the web-based consultation. Furthermore, parents of technology users asked for support more frequently than nontechnology users on diabetes nannies and flexible working hours (both significant at *p* < 0.0001).

Comparing the high- and low-burden category groups showed that the high-burden category groups were more welcoming of all diabetes support compared to the low-burden category groups in general (Table 4). All three types of the high-burden groups asked for significantly more help on self-help/patient groups, diabetes nannies, care services, and flexible working hours compared to their low-burden category counterparts. The high daily and emotional burden groups asked for additional support for home office opportunities.

## 4. Discussion

The present study examined different categories of diabetes care burden: daily, emotional, and physical in parents of children with T1D in a large sample of 1,107 families from nine pediatric diabetes sites in Germany. One of the main findings of the current study was that the emotional burden was the highest compared to daily and physical burdens. Our finding corroborates other studies, which reported that parents' worries about the future and long-term complications contributed significantly to their emotional burden [5, 6, 29]. Collecting information on three different types of burdens uncovered the uniqueness of emotional burdens compared directly to daily and physical burdens in parents.

Examining the associations of burdens with demographic and clinical features revealed that mothers reported significantly higher burdens than fathers. This finding is comparable to the previous study reporting mothers are more likely than fathers to experience occupational strains due to the diagnosis of T1D in their child [21]. Our finding is also congruent with the report from a Norwegian population-based survey examining an age-comparable sample as in our study. Mothers experience significantly greater stress and emotional distress related to T1D in their children than fathers [19].

The physical burden was the lowest among parents and was only marginally different by the reporting parent's gender (*p* = 0.024). However, the living arrangement made a difference in how much physical burden parents, specifically mothers, experienced. This finding provides support for recent studies revealing a significant association between family structure and glycemic control [13, 30]. This is particularly important because of the increasing trend of one-parent households in recent years, and in most cases, the mother lives with the child. This sole responsibility of caring for a child with diabetes among single mothers augments the physical load. Single parenting is known to increase parental stress [31, 32], and this group of single parents needs additional support to reduce the physical demands of caring for a child with diabetes if the responsibilities cannot be shared domestically.

Another interesting finding was that some parental burdens diminished with a child's age. Parents of older children reported less daily and physical strain in our study. Perhaps, it is because, with the increasing age of the child, parents are less involved in daily diabetes management [33, 34]. Aging involves gaining independence and assuming the responsibility of diabetes self-management thereby reducing parents' day-to-day tasks, reflected in reduced daily and physical strains. This specific association between the age of the child and the caregiving burden has been also previously reported [18]. However, as our results indicate, parents' worries and fears about their child's future do not diminish with the child's increased autonomy. As the child experiences independence and often wants to take over responsibility [35, 36], the mothers who created a very close caregiver-receiver relationship for many years may find it particularly difficult to adapt to the change and reduce responsibility. In some cases, giving up involvement in diabetes therapy in their child is associated with decreasing metabolic control in adolescents [37]. This can in turn contribute to parental worries and emotional burdens.

The finding on the effects of youth age on different parental burdens is of particular significance because it has not been previously reported in a large sample with a wide age range of children. Studies often examine a specific age segment of patient groups, either preschool-age children or teenagers due to differences in their developmental stage. However, including children of all age groups in this study permitted us to examine the effect of age and reveal that parents' emotional burdens, while being high, do not diminish over time. Left unchecked, long-lasting emotional strains not only reduce the quality of life and increase mental health problems in parents but also negatively impact the child's well-being and diabetes management [4, 7, 38].

Technology use was linked to high daily and physical caregiving burdens. The use of diabetes technology leads to several additional tasks for parents to accomplish [22], and these additional tasks may have contributed to a heightened sense of daily and physical responsibilities. Evidence suggests that the successful use of diabetes technology contributes to lower HbA1c [23]. In this study, lower HbA1c levels were also associated with CGM use in children. Nevertheless, concurrent high levels of daily and physical loads reported by parents are disconcerting and should be addressed in diabetes education. Since 2018, more advanced diabetes technology such as automated insulin delivery (AID) systems has become available for young people with diabetes. Reduction of diabetes management burden in parents has been reported for the use of closed-loop systems [39]. However, whether reduced diabetes management burdens can also reduce the overall emotional burden on parents is unclear. Further research is needed to determine how these decreased demands associated with advanced technology use would affect different aspects of parental burdens.

In a regression model examining additional sociodemographic factors for three burdens, parents' immigrant status and being a single parent were significant risk factors for a high physical burden. This may be in part explained by a lack of practical everyday assistance for those parents from their family or spouse in close proximity. Similar to our study results, recent data from large international registries have also shown that access to diabetes technology is associated with migration background [40]; therefore, even general access to diabetes care support outside the home could be insufficient in these families.

The level of glucose control (HbA1c) was the only predictor for emotional burden aside from the gender of the reporting parent. Parents' concerns about acute and long-term complications may rise and fall with changing glycemic control. The use of technology did not add emotional strain nor reduce it. Interestingly, no other sociodemographic variables other than parents' gender were linked to the emotional burden. It is likely that other parameters exist but were not included in this study. Perhaps certain psychosocial factors such as the cognitive appraisal of stress, stress management style, and coping strategies may better predict the parent-reported emotional burden [2]. Additional research must explore these psychosocial parameters to uncover the risk factors associated with emotional burdens in parents, and concurrently, efforts to reduce emotional burdens are needed.

Approximately 15% of mothers in the current study reported that they were diagnosed with a mental disorder since the diagnosis of diabetes in their child. Considering this number does not include mothers with preexisting mental health disorders or subclinical cases, an alarming number of mothers exhibited emotional difficulties caring for their children with diabetes. While there is a focus on psychosocial care for children with diabetes, there is a lack of appropriate stepwise interventions for those parents with a high level of emotional burden who do not meet the criteria for a mental health disorder in the German health care system. Short and low-threshold psychological interventions might fill this gap, and the benefit of these interventions should be examined.

The ways to reduce parental diabetes distress were measured in the wishes and needs for support. Almost all parents asked for further support in the ongoing care of their children with diabetes, especially in the form of diabetes training. The majority of parents (78%) wanted more diabetes training across all types and levels of burdens. This is consistent with the findings from a recent study that parents wanted more diabetes education [5]. Practical skills training are a perfect platform to target specific education needs, such as the use of diabetes technology in everyday life with a young child [22, 41–43]. Our results also suggest that parents want more regularly offered personalized diabetes face-to-face training rather than web-based consultations. Others have also found that parents prefer support from their personally known health care providers who have a deeper understanding of the child and the unique family situation [5]. As the study was conducted before the COVID-19 pandemic, parents presumably had less experience with online education and consulting. Future studies may show other preferences. On the other hand, training sessions in a group format can also fulfill parents' need to interact confidentially with other families in a similar situation. Additional studies are warranted to explore the most efficacious forms of diabetes self-management education.

In our study, parents also wanted support from work such as flexible working hours and home office opportunities, which requires reorganization of work and the social environment at large. The caregiving responsibilities affect women more severely than men. As evidenced in a previous study, mothers have shown commendable sacrifice to accept additional responsibility by compromising their current and future financial and occupational situations [21]. Supporting these families will not only provide relief for these mothers but also reduce social inequality among members of our community.

Since conducting this study, due to the break of COVID-19, home office and flexible working hours have been largely implemented. During this time, parents reported high caregiver burdens, which were linked to family conflict and depressive and anxiety symptoms in families with and without children with diabetes [44, 45]. This indicates that providing home office or flexible hours alone may not reduce distress, but rather enhancing parents' ability to effectively take care of their children is crucial. Considering this, the structured diabetes training sessions offered regularly would play a pivotal role to supply parents with necessary skills in dealing with situations of high demands.

There are several limitations to this study. The results are obtained from a large sample of families from nine qualified pediatric diabetes centers in Germany. Whether these findings can be replicated in general in other groups remains to be clarified. As seen in most of the caregiver literature, the number of fathers engaging in this study was small because the majority of caregivers are mothers. We have statically controlled for the sample size differences during our analyses by randomly selecting a group of mothers to be compared to fathers in sensitivity analyses to confirm the robustness of our primary findings. Still, an equal number of fathers and mothers selected at random would have been preferable [5, 8]. Also, our study did not include an appropriate control group such as families with a child with other diseases or no diseases. Another area for improvement was that the missing data and accuracy of data could not be controlled and verified because the survey was self-administered in paper format and anonymous. A thoughtfully designed online survey with mandatory fields and answer range options could circumvent these limitations. Moreover, because parents who could not give verbal consent or complete the questionnaire in German were excluded, the generalizability of our findings may be limited. Lastly, the level of three parental burdens in this study was assessed via a global rating. Therefore, it was not possible to describe in detail, what components of daily, emotional, or physical burdens were most stressful for parents.

Parenting, in general, is a long-term project, which is deeply fulfilling, challenging, and exhausting, especially when caring for a child with T1D. When parents are exhausted and burdened, it is difficult to be a good role model or offer the appropriate and necessary support for their child using positive parenting strategies. Considering the influence of parents' well-being on the children's glycemic control, diabetes treatment should provide support for the entire family to meet their everyday demands in caring for the child with diabetes. Practical efforts to reduce parental burdens including assessments for the early detection of symptoms and interventions for clinical and subclinical mental health issues are urgently needed to provide continuous support for parents of children with diabetes.

## Figures and Tables

**Table 1:** Characteristics of 1,107 German parents living with a child with type 1 diabetes.

Participating parent characteristics	Mean (SD) or *n* (%)	
	Mothers	Fathers
	*n* = 919 (83.0%)	*n* = 188 (17.0%)
Current age, years	43.9 (6.5)	46.0 (6.9)
Born in Germany	774 (84.7%)	155 (83.8%)
Professional qualification		
University degree (associate degree and above)	278 (30.6%)	88 (47.3%)
Apprenticeship or traineeship	540 (59.5%)	84 (45.2%)
Other or no qualification	89 (9.8%)	14 (7.5%)
Living arrangement		
With a spouse	725 (78.9%)	173 (92.0%)
Alone	194 (21.1%)	15 (8.0%)
Number of children		
One child	231 (25.1%)	40 (21.3%)
Two	444 (48.3%)	86 (45.7%)
Three	172 (18.7%)	45 (23.9%)
Four or more	72 (7.8%)	17 (9.1%)
Youth characteristics		
Current age, years	12.7 (3.9)	
Gender (female/male)	501(46.7%)/571(53.3%)	
Age at type 1 diabetes diagnosis, years	6.7 (3.6)	
Youth clinical characteristics		
Disease duration, years	5.9 (3.8) years	
Most recent HbA1c, %/mmol/mol	7.67 (1.14)/60.27 (12.49)	
Current use of CSII, yes	721 (65.4%)	
Current use of CGM^†^, yes	198 (39.5%)	

Note: the % is calculated excluding missing values. ^†^Total *n* = 672.

**Table 2:** Median and mean (SD) of daily, emotional, and physical parental burdens by parent's gender, living arrangement, child's age, and technology use.

	Daily burden			Emotional burden			Physical burden		
	Mdn	*M* (SD)	*p*	Mdn	*M* (SD)	*p*	Mdn	*M* (SD)	*p*
Gender of the participating parent			<0.0001			<0.0001			=0.024
Mothers	4	3.48 (1.09)		4	3.72 (1.10)		2	2.51 (1.27)	
Fathers	3	3.00 (1.01)		3	3.19 (1.14)		2	2.25 (1.04)	
Parent's living arrangement			=0.021^†^			=0.036^†^			=0.002^†^
With a spouse (*n* = 898)	3	3.40 (1.09)		4	3.60 (1.14)		2	2.41 (1.21)	
Mother (*n* = 725)	4	3.45 (1.10)		4	3.70 (1.11)		2	2.45 (1.25)	
Father (*n* = 173)	3	2.98 (1.01)		3	3.17 (1.15)		2	2.24 (1.04)	
Alone (*n* = 209)	4	3.56 (1.04)		4	3.79 (1.07)		3	2.72 (1.32)	
Mother (*n* = 194)	4	3.58 (1.04)		4	3.81 (1.07)		3	2.75 (1.34)	
Father (*n* = 15)	3	3.27 (0.96)		3	3.47 (1.06)		3	2.33 (0.98)	
Child's age group			<0.0001			=0.095			<0.0001
<13 years (*n* = 488)	4	3.63 (1.03)		4	3.70 (1.15)		3	2.73 (1.25)	
≥13 years (*n* = 577)	3	3.21 (1.08)		4	3.61 (1.08)		2	2.27 (1.18)	
Diabetes technology: CSII, CGM			<0.0001			= 0.114			<0.0001
No use of CSII or CGM (*n* = 221)	3	3.11 (1.14)		4	3.54(1.20)		2	2.21 (1.26)	
Use of ≥ 1 technology (*n* = 744)	4	3.53 (1.05)		4	3.70 (1.08)		2	2.56 (1.24)	

Note: the *p* values are based on the Mann–Whitney test. ^†^According to the Mann–Whitney test of two living arrangement groups (living with a spouse vs. alone).

**Table 3:** Three exploratory logistic regressions of daily, emotional, and physical burdens of parents living with a child with type 1 diabetes.

High daily burden	*B* (SE)	*p*	*b*	95% CI
Child's current age	-0.15 (0.03)	< 0.0001	0.86	0.82-0.91
HbA1c	0.22 (0.07)	0.001	1.25	1.10-1.44
Mothers	1.10 (0.22)	< 0.0001	3.00	1.97-4.58
Parent's current age	0.04 (0.02)	0.008	1.04	1.01-1.07
Diabetes technology use (≥1)	0.44 (0.18)	0.017	1.55	1.08-2.21
High emotional burden	*B* (SE)	*p*	*b*	95% CI
HbA1c	0.15 (0.07)	0.032	1.16	1.01-1.32
Mothers	0.92 (0.20)	<0.0001	2.51	1.71-3.69
High physical burden	*B* (SE)	*p*	*b*	95% CI
Child's current age	-0.11 (0.03)	< 0.0001	0.90	0.85-0.95
Mothers	0.93 (0.30)	0.002	2.53	1.41-4.54
Not born in Germany	0.93 (0.24)	< 0.0001	2.52	1.59-4.00
Parent's current age	0.04 (0.02)	0.016	1.04	1.01-1.08
Living arrangement/single parent	0.63 (0.22)	0.004	1.88	1.23-2.88
Diabetes technology use (≥1)	0.47 (0.24)	0.045	1.61	1.01-2.56

Note: burden measures were dichotomized as high (“high” and “very high”) or low (“very low,” “low,” and “moderate”) for logistic regression. Daily burden, *n* = 800: *R*^2^(Nagelkerke) = 0.120, model *χ*^2^(5) = 75, 33, *p* < 0.0001. Emotional burden, *n* = 802: *R*^2^(Nagelkerke) = .045, model *χ*^2^(2) = 27, 20, *p* < 0.0001. Physical burden, *n* = 798: *R*^2^(Nagelkerke) = .090, model *χ*^2^(6) = 48, 13, *p* < 0.0001. Ten independent variables were the child's current age, HbA1c, disease duration, and technology use and parents' gender, age, education, living arrangement/single parent, immigrant status, and the number of children. Because of the exploratory nature of the analysis, the significance level was set at *p* < 0.05.

**Table 4:** Frequency of diabetes support requested by all parents and by low- and high-burden parents living with a child with type 1 diabetes.

	All *N* = 1,014	Daily burden		*p*	Emotional burden		*p*	Physical burden		*p*
		Low	High		Low	High		Low	High	
		*n* = 495	*n* = 506		*n* = 404	*n* = 600		*n* = 783	*n* = 224	
Diabetes training	77.7%	75.4%	79.4%	0.131	74.5%	79.5%	0.065	76.5%	80.8%	0.204
Self-help group	23.3%	16.0%	29.8%	<0.0001	14.6%	28.8%	<0.0001	21.2%	30.8%	0.003
Diabetes nannies	37.5%	25.1%	50.0%	<0.0001	26.0%	45.3%	<0.0001	31.5%	58.0%	<0.0001
Web-based consultation	41.6%	40.4%	43.1%	0.405	42.3%	40.8%	0.648	42.1%	40.2%	0.645
Website for parents^†^	53.4%	51.1%	55.5%	0.164	54.2%	52.2%	0.562	53.1%	54.9%	0.649
Care service	18.6%	11.9%	25.5%	<0.0001	12.1%	22.8%	<0.0001	15.5%	29.5%	<0.0001
Home office opportunities	43.9%	37.2%	50.8%	<0.0001	37.4%	47.8%	0.001	42.0%	49.6%	0.047
Flexible working hours	61.2%	53.3%	69.2%	<0.0001	54.7%	65.3%	0.0007	58.9%	68.8%	0.008

Note: burden answers were dichotomized: low category includes “very low,” “low,” and “moderate”; and high category includes “high” and “very high.” Significance level according to *χ*^2^ analyses. ^†^Website for parents of children with diabetes.

## Data Availability

The data are available from the corresponding author upon reasonable request.
